# Fecal Microbiota Transplantation: A Microbiome Modulation Technique for Alzheimer’s Disease

**DOI:** 10.7759/cureus.16503

**Published:** 2021-07-20

**Authors:** Varsha Nandwana, Shibajee Debbarma

**Affiliations:** 1 Medicine, Lady Hardinge Medical College and Associated Hospitals, New Delhi, IND; 2 Community Medicine, Lady Hardinge Medical College and Associated Hospitals, New Delhi, IND

**Keywords:** alzheimer’s disease, gut microbiota, dysbiosis, fecal microbiota transplantation, gut-brain axis

## Abstract

Alzheimer’s disease (AD) is the most common form of dementia and the fifth leading cause of death among the elderly. AD involves parts of the brain that can lead to progressive memory loss and impaired language skills and cognitive thinking, affecting one’s ability to carry out daily activities. Aging, bad dietary habits, family history, as well as altered gut microbiota composition may play a role in the pathogenesis of AD. Although the association between the imbalance of gut microbiota and AD is still difficult to determine, it has been suggested that dysbiosis can lead to the increased secretion of lipopolysaccharides and amyloid, which may impair the permeability of the intestine and the blood-brain barrier. Moreover, it can progress the process of neuroinflammation, amyloid-beta formation, and ultimately neuronal death. Microbiota-targeted interventions such as personalized diet, probiotics, or fecal microbiota transplantation (FMT) might represent a potential therapeutic option for AD. This review article discusses the procedure of FMT and its possible side effects on the recipient’s body. In addition, we review the role of FMT in the context of its application in various nervous system-related disorders (AD, Parkinson’s disease, multiple sclerosis).

## Introduction and background

The gut microbiota plays a key role in modulating the gut-brain axis, which is a bidirectional communication network that involves the central nervous system, the autonomic nervous system (sympathetic and parasympathetic branches), the enteric nervous system, and the hypothalamic-pituitary-adrenal axis [1]. Recent advances have revealed that the microbiota of the human gut has numerous beneficial functions, such as immune system development, resistance to pathogens, vitamin synthesis, production of metabolites such as short-chain fatty acids (SCFAs), nutrient and drug metabolism, and maintenance of the structural integrity of the intestinal mucosal barrier [2]. In humans, dysbiosis and changes in gut microbiome composition have been found to contribute to inflammatory bowel disease, type 2 diabetes, metabolic syndrome, obesity, colorectal cancer, Alzheimer’s disease (AD), and numerous other diseases [3]. AD is a disastrous neurological disorder affecting 5.8 million Americans (aged 65 years or older) in 2020. This number is expected to increase to 14 million by 2060. As the prevalence of AD increases, the total estimated worldwide cost of dementia is also expected to increase to US$2.0 trillion by 2030 [4]. AD was the sixth most common cause of death in 2017, accounting for 121,404 deaths in the United States, and the fifth most common cause of death among elderly Americans (65+ years) [5]. The sizeable economic burden of AD, as well as its growing prevalence, are leading researchers to look for preventive or disease-modifying treatments. There are various gut microbiota modulation interventions such as diet modification, prebiotics, probiotics, synbiotics, or fecal microbiota transplantation (FMT). FMT includes the transplantation of the gut microbiota from a donor to a recipient to refurbish the intestinal microflora of the recipient. It has been proven to be a successful treatment for recurrent *Clostridium difficile* infections [6]. In this review, we summarize the procedure of FMT and its application in the treatment of various neurological disorders with a special emphasis on AD.

## Review

Dysbiosis and Alzheimer’s disease

**Figure 1:**
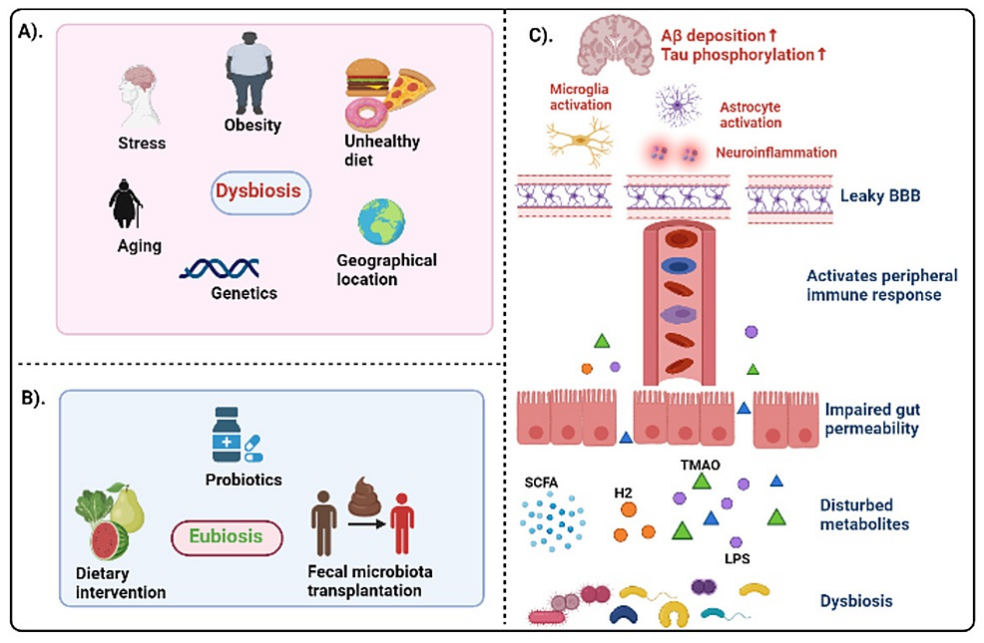
(A) Various factors influencing dysbiosis, that is, alteration of gut flora. (B) Various microbiota modulation techniques inducing eubiosis, that is, restoration of altered gut composition. (C) Schematic diagram representing the interplay between dysbiosis and AD pathology. Created with BioRender.com. Aβ: amyloid-beta; AD: Alzheimer’s disease; BBB: blood-brain barrier; LPS: lipopolysaccharide; SCFA: short-chain fatty acid; TMAO: trimethylamine N-oxide

In Western countries, more than 40 million elderly people suffer from AD, accounting for over 65% of all dementia cases. The increased life expectancy of the global population has led to an increase in age-related diseases, including AD [7]. The primary neuropathological criteria for AD diagnosis include the presence of misfolded amyloid-beta (Aβ) plaques and deposition of hyperphosphorylated tau as neurofibrillary tangles (NFT) [8]. Although the greatest known risk factor for AD is increasing age, multiple factors such as genetics, family history, lifestyle, and environment may influence the onset of AD. There is increasing evidence suggesting the linkage between microbes that naturally reside in the body and the development of many of the hallmark features of AD, including Aβ plaques and NFT [9]. It has been shown that the gut microbiota plays a crucial role in some brain processes such as myelinization, microglial activation, and neurogenesis, which is closely related to behavioral, mood, and cognitive modulation [10]. In addition to aging, unhealthy diet, stress, or obesity, several factors that affect gut microbiota composition are associated with AD [11]. The impaired gut microbial composition alters the expression of tight junction proteins, that is, zonulin and occludin, in epithelial colon cells. Intestinal permeability is increased due to problems in tight junction competence. The impaired gut microbial composition can increase lipopolysaccharides, amyloids, and trimethylamine N-oxide, and simultaneously decrease the beneficial metabolites such as SCFA and hydrogen [11]. Toll-like receptors (TLR) 2 and 4 are activated by exposure to enteropathogenic bacteria. This results in nuclear factor kappa B (NFkB) activation and secretion of pro-inflammatory cytokine interleukin-12 (IL-12). When IL-12 is increased, T-helper (Th)1/Th2 immune responses improve accompanied by an increase in tumor necrosis factor (TNF), interferon-gamma (IFN-γ), and IL-6 [12]. Consequently, elevated levels of circulating pro-inflammatory cytokines could enter the central nervous system through the leaky blood-brain barrier (BBB) (due to disrupted endothelial tight junction), leading to the translocation of blood-borne immune cells such as macrophages and neutrophils. In addition, as a result of inflammatory mediators such as cytokines and microbes, or bacterial products, microglia become activated, causing the breakdown of the extracellular matrix and dysfunction of astrocyte, pericyte, and neuronal cells [13]. This cascade acts as a trigger for Aβ and tau accumulation, leading to neurodegeneration and progression of AD pathology (Figure 1).

During the course of recent research, Cattaneo et al. indicated that cognitive impairment and amyloid deposition in the brain was associated with an increase in the abundance of pro-inflammatory taxa *Escherichia/Shigella* and reduced anti-inflammatory taxon *Eubacterium rectale* [14]. In a healthy individual, two phyla, *Firmicutes* and *Bacteroidetes*, represent about 90% of the gut microbiota, while the rest is composed of *Actinobacteria*,* Proteobacteria*,* Fusobacteria*,and* Verrucomicrobia* [15]. Vogt et al. examined fecal samples from AD patients and compared them with controls (age and sex-matched individuals). According to the study, *Firmicutes* and *Bifidobacterium* are less abundant in the AD microbiome whereas *Bacteroides* are more abundant. In addition, they observed a correlation between the relative abundance of bacterial genera and the levels of cerebrospinal fluid biomarkers of AD [16]. According to Bäuerl et al., a transgenic APP/PS1 (Tg) mouse model was also successful in showing how AD pathology shifted gut bacterial profiles in favor of *Proteobacteria* and *Erysipelotrichaceae*, which potentially contributed to disease progression and severity [17]. Cox et al. demonstrated that *Bacteroides* colonization worsens Aβ deposition, while long-term calorie restriction may alter the gut environment and inhibit the cultivation of microbes that contribute to age-related cognitive decline [18].

Potential therapeutic strategies for Alzheimer’s disease

As it is already known that dysbiosis is strongly correlated with the development of neurodegenerative diseases, gut microbiota modification can also play a key role in combating AD. Most therapies for AD simply delay the loss of cognition and memory as there is no definitive treatment for AD. Several recent studies have pointed toward the role of the human microbiome in regulating multiple neurochemical pathways through the gut-brain axis [19]. It has been shown that plant-based diets, omega-3 polyunsaturated fatty acids, antioxidants, and reducing dietary saturated fats, animal-derived proteins, and refined sugars reduce the risk of cognitive decline and early onset of AD [20]. Diet-based therapeutic interventions include calorie restriction, prebiotic-rich diet, probiotic supplementation, probiotic-enriched diet, and polyphenol supplementation [19].

The probiotic bacterium helps promote the health of the recipient, while the prebiotic bacterium is primarily a food source for the probiotic bacterium [21]. A study by Bonfili et al. treated a transgenic mouse model of AD with a cocktail of *Lactobacillus *(*L. acidophilus*,* L. brevis*,* L. delbrueckii *subsp. *bulgaricus*,* L. paracasei*,* L. plantarum*) and *Bifidobacteria* (*B. breve*, *B. longum*,* B. infantis*) called SLAB 51 for a duration of four months. The increase in *Bifidobacterium* spp. and the reduction in *Campylobacterales* spp. observed in AD mice upon administration with SLAB51 as well as the altered content of metabolites of intestinal bacteria such as SCFA improved cognitive functions [22]. A study by Ho et al. reported that intestinal microbiota act to protect against AD in part by enhancing the formation of select SCFAs that inhibit the formation of soluble aggregates of Aβ [23]. In one study, Kobayashi et al. showed that *B. breve* A1 consumption decreased inflammatory and immune-reactive genes expressed in hippocampal neurons [21]. In a recent study, Abraham et al. showed that probiotics were sufficient to improve cognitive performance in mice with AD compared to mice not treated with probiotics [24].

In addition to dietary intervention and probiotics, FMT is the most effective therapeutic option that modulates the gut microbiome. This approach has shown improvements in various neurological disorders such as AD, Parkinson’s disease (PD), and multiple sclerosis (MS).

Fecal microbiota transplantation

Historical Applications of Fecal Microbiota Transplantation

Approximately 1,700 years ago, when human fecal material was called “yellow soup,” the concept of FMT was introduced by a Chinese medical scientist named Ge Hong when he administered the “soup” orally to treat patients with severe diarrhea or gastroenteritis [25]. Christian Paullini, a German-born scientist, was the first to demonstrate the curative potential of human excretion with his work on “Heilsame Dreck-Apotheke,” that is, “healing mud pharmacy” [26]. The first human fecal enemas were used to treat pseudomembranous colitis by Eiseman et al. in 1958 using four case studies [27]. Borody et al. performed “an exchange of bowel flora” in a patient with refractory ulcerative colitis showing full and lasting clinical recovery after treatment [28]. In 2013, van Nood et al. noticed that, in patients with recurrent *C. difficile* infection, the introduction of donor feces had more efficacy in comparison to antibiotics alone [29].

Procedure for Fecal Microbiota Transplantation

**Figure 2:**
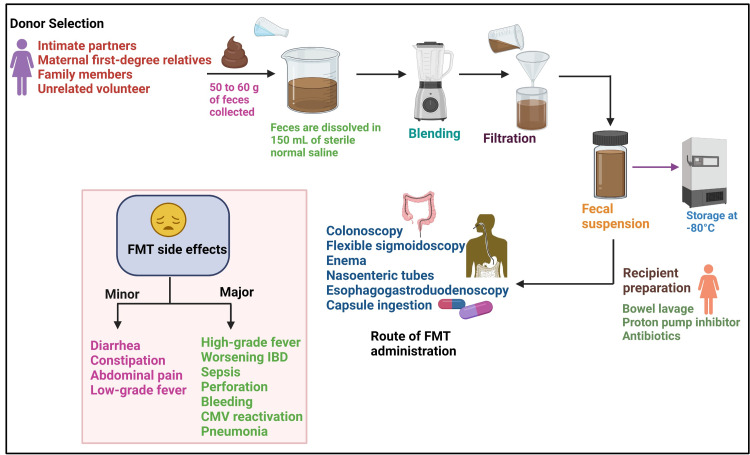
The schematic diagram of the FMT procedure and the associated adverse events. Created with BioRender.com. CMV: cytomegalovirus; FMT: fecal microbiota transplantation; IBD: inflammatory bowel disease

The donors for FMT can be selected from intimate partners, family members, or unrelated volunteers (Figure 2) [30]. The following conditions are generally considered to prevent donor recruitment: taking antibiotics within three months; being on immunosuppressive or chemotherapy agents; acquiring HIV or hepatitis B or C recently; being obese, having inflammatory bowel disease (IBD) or irritable bowel syndrome; having an underlying infection, GI malignancy, polyposis, high-risk sexual behaviors, and use of illicit drugs; and having a history of recent incarceration or travel to areas with endemic diarrhea [31,32]. A potential donor with an acceptable medical history needs to be screened for infective diseases by undergoing blood and stool tests [33]. Nowadays, due to the coronavirus disease 2019 pandemic, it has been suggested to rule out severe acute respiratory syndrome coronavirus 2 infection in all donors through nasopharyngeal polymerase chain reaction and RNA detection in rectal swab [34]. A potential donor can provide fresh stool within one month after screening. The stool is collected and sealed in a clean opaque plastic bag before placing it in a larger storage bag [32]. Though the ideal amount of stool weight to be used for FMT has not been standardized, approximately 50 to 60 g of stool is recommended for each treatment. The stool can be stored for up to eight hours at 4°C as after eight hours at 4°C or room temperature, bacterial survival declines. The feces are dissolved in approximately 150 mL of normal saline by hand stirring or by using a blender [35]. The mixture is then filtered through a sterile gauze pad or steel strainers to remove large particulate matter [36]. Thereafter, the suspension is drawn into 60 mL catheter tip syringes. Furthermore, the final fecal material should be clearly labeled and stored at -80°C. On the day of the infusion, it should be thawed in a warm water bath at 37°C and infused within four hours from thawing [32] (Figure 2).

FMT can be administered directly to the colon using colonoscopy (efficacy of 84-93%) [37] and less frequently through flexible sigmoidoscopy or an enema [38]. For patients with ileus, severe colitis, or contraindication to colonoscopy, FMT can be provided through the upper GI tract via nasoenteric tubes, esophagogastroduodenoscopy, or capsule ingestion [38] (Figure 2). Capsule delivery is the most recent modality of FMT, which seems to be favorable for those who are opposed to lower GI tract access and are geographically distant from an institution that performs colonoscopy [39]. Before an FMT, the patient must be vigorously prepared so that no residual stool is present [35]. If the fecal solution is administered via colonoscopy, the recipient usually undergoes a bowel preparation to flush out the pre-existing bacteria. When a nasogastric tube is used to administer the fecal sample, a proton pump inhibitor may be administered in advance to increase the survival of the transplanted bacteria [40] (Figure 2).

Adverse Events of Fecal Microbiota Transplantation

In general, FMT is a safe and efficacious therapeutic method with limited side effects. Many recent large studies have confirmed that minor adverse events including diarrhea, constipation, abdominal discomfort, bloating, and transient low-grade fevers are the most likely to occur and are likely to resolve within days to weeks. Uncommon severe side effects such as high-grade fever, worsening IBD activity, infection and sepsis, the transmission of enteric pathogens, perforation, bleeding, cytomegalovirus reactivation, and pneumonia [41] are associated with endoscopy and sedation (Figure 2). It has been reported in some case reports that there might be some abeyant connection between FMT and certain conditions such as rheumatoid arthritis, idiopathic thrombocytopenic purpura, and peripheral neuropathy [42]. FMT recipients should be informed about the potential adverse effects before the procedure.

Fecal microbiota transplantation as a restorer of altered gut microbiota in Alzheimer’s disease

Restoring gut microbial composition and reducing dysbiosis by FMT could be an effective approach for various neurological disorders, but the evidence is limited. In an experiment conducted by Zhanet al., mice were first treated with broad-spectrum antibiotics for 14 consecutive days. Subsequently, the authors transplanted fecal bacteria from senescence-accelerated mouse prone 8 (SAMP8) or senescence-accelerated mouse resistant 1 (SAMR1) mice into pseudo-germ-free mice and noticed that pseudo-germ-free mice that received fecal bacteria transplants from SAMR1 mice but not from SAMP8 mice showed improvements in spatial learning and memory [43]. Yu et al. used diabetic mice with and without cognitive deterioration and performed fecal bacteria transplantation from noncognitive dysfunction mice into the gut of pseudo-germ-free mice. They noticed a significant improvement in host Morris water maze performance indices, an effect linked with change in β-diversity and relative abundance of host intestinal microbes [44]. Furthermore, Dodiya et al. demonstrated that fecal microbiota transplantation from age- and sex-matched AD male mice without antibiotics into antibiotic-treated AD male mice reinstituted the intestinal bacteria and partially refurbished Aβ pathology and microglial morphology. They observed relatively higher expression of anti-inflammatory cytokines such as IL-10 and lower expression of pro-inflammatory cytokines including IL-1B, IL-2, IL-3, IL-17A, LIX (CXC5), RANTES (CCL5), and the cluster of differentiation 30 (CD30) and CD40 in antibiotic-treated male mice. [45]. Fuji et al. created a humanized mice model by transplanting germ-free mice with a fecal sample from a healthy person and an AD patient. They noticed that gut microbiota transplanted from an affected patient affected mouse behavior remarkably. Moreover, lower metabolites related to the nervous system, including γ-aminobutyrate, taurine, and valine, in the feces of mice transplanted with microbiota from AD patients has been noted [46]. Recently, using APPswe/PS1dE9 transgenic (Tg) mouse model, Sun et al. showed that FMT can reduce the brain deposition of Aβ and decrease the phosphorylation of tau protein and levels of Aβ40 and Aβ42. In addition, improvement in cognitive function and an increase in synaptic plasticity have also been observed in Tg mice, thus demonstrating FMT as a potential therapeutic strategy for AD [47]. The case of an 82-year-old AD patient was reported by Hazan in 2020, who received a single FMT from his 85-year-old wife, but his symptoms of *C. difficile* infection resolved within two months following the test. In addition, after two months of FMT, the Mini-Mental State Examination (MMSE) score of the patient increased from 20 (mild cognitive impairment) to 26 (normal cognitive function), which further improved (MMSE score 29) after four months of follow up [48]. Holsinger et al. using the 5XFAD mouse model described an improvement in spatial and recognition memory and reduction in amyloid pathology after seven days of FMT from wild-type donor to 5XFAD mouse, thus exhibiting the efficacy of FMT as a therapeutic in AD [49].

Fecal microbiota transplantation in various other neurological disorders

PD is a progressive nervous system disorder that affects predominately dopamine-producing neurons in a specific area of the brain called substantia nigra. PD patients may experience resting tremor, bradykinesia, limb rigidity, gait, and balance problems. Sun et al. demonstrated that gut microbial dysbiosis is involved in PD pathogenesis using a PD mouse model. FMT from healthy mice improved motor function, increased striatal neurotransmitters, and decreased neuroinflammation [50]. Another study by Zhou et al. noticed increased dopamine levels in PD mice that had received FMT from normal mice with fasting-mimicking diet treatment to antibiotic-pretreated PD mice [51]. MS is a demyelinating disorder in which the immune system attacks the protective covering of nerves, thus disrupting the communication between the brain and the body. Patients with secondary progressive MS as well as concomitant recurrent *C. difficile* infections were treated with a single FMT. The FMT resolved the recurrent *C. difficile* infections and was suggested to prevent MS disease progression for over 10 years [52]. Li et al. conducted a study using experimental autoimmune encephalomyelitis, a mouse model of MS. They observed that FMT can correct altered gut microbiota and confer protection on the BBB, myelin, and axons, as well as can reduce the activation of microglia and astrocytes, thus suggesting the role of FMT as a potentially effective treatment for MS [53].

## Conclusions

The composition of healthy gut microbiota may change due to nutrition, medications, lifestyle, geographical location, stress, and dietary habits. An imbalance in the intestinal bacteria can influence the onset and progression of AD. Thus, modulation of gut microbiota using dietary intervention, probiotics, and FMT represents a forward-looking approach for various neurological disorders such as AD, PD, and MS. Currently, the rationale for the clinical application of FMT is based on a small number of case reports and animal models. Increasing evidence suggests that the capsular form containing desirable bacteria is a more comfortable option for patients and may replace FMT in the future. For a complete understanding of the role of FMT in neurological diseases, we need a large-scale, randomized, double-blind trial.
